# Simultaneous Cardiopulmonary Exercise Testing and Echocardiography for Investigation of Cardiopulmonary Dysfunction in Outpatients: Protocol for a Scoping Review

**DOI:** 10.2196/52076

**Published:** 2024-02-12

**Authors:** Benjamin Gerhardy, Shanthosh Sivapathan, Sam Orde, Lucy Morgan

**Affiliations:** 1 Department of Respiratory Medicine Nepean Hospital Kingswood Australia; 2 Department of Intensive Care Medicine Nepean Hospital Kingswood Australia; 3 Faculty of Medicine and Health Sciences University of Sydney Kingswood Australia; 4 Department of Respiratory Medicine Concord Repatriation General Hospital Concord Australia

**Keywords:** cardiopulmonary, echocardiography, exercise, cardiopulmonary exercise test

## Abstract

**Background:**

Cardiopulmonary dysfunction is a complex process with a broad range of etiologies. Investigations performed either at rest or those that only assess the function of a single organ (heart or lungs) are often insufficient. A simultaneous cardiopulmonary exercise test with stress echocardiography is a new approach to assessing cardiopulmonary dysfunction as it provides anatomical and functional imaging simultaneously while under increasing stress. To date, the application of cardiopulmonary exercise test-stress echocardiography (CPET-SE) has been broad and without structure, and its effect on patient outcomes is unclear.

**Objective:**

The objective of this scoping review is to explore and analyze the evidence regarding the role of simultaneous CPET-SE in investigating cardiopulmonary dysfunction in outpatients. It will include any published study in which adult (older than or equal to 18 years of age) patients have completed a CPET-SE for the investigation of cardiopulmonary dysfunction.

**Methods:**

This review will follow the Arksey and O’Malley framework, supported by the Joanna Briggs Institute methodology for scoping reviews. It will use the PRISMA-ScR (Preferred Reporting Items for Systematic Reviews and Meta-Analyses Extension for Scoping Reviews) checklist. Data sources will include MEDLINE, Scopus, Embase, and Cochrane (including reviews, trials, and protocols) electronic databases, with no date range defined. The search will be limited to the English language with no restrictions regarding pathology. Secondary references of the included sources will also be assessed by a hand search for suitability. A 2-person title-abstract screen and data charting process will be used. Independent experts will be used for consultation including an academic librarian and clinicians. The Covidence software will be used for article screening.

**Results:**

This scoping review will provide a unified and detailed description of the applications of CPET-SE in investigating cardiopulmonary dysfunction. This will provide a platform for future research harnessing this investigatory method. The results will be presented in both tabular and graphical formats to ensure clarity. The results of this scoping review will be submitted to a relevant peer-reviewed academic journal for publication.

**Conclusions:**

The CPET-SE is a powerful tool for investigating cardiopulmonary dysfunction but remains in its infancy with a patchwork approach to indications, data reporting, and interpretation. This scoping review will unify the literature and provide a platform for future researchers and the development of a comprehensive application guideline.

**Trial Registration:**

Open Science Framework; https://osf.io/98r3e

**International Registered Report Identifier (IRRID):**

PRR1-10.2196/52076

## Introduction

Cardiopulmonary dysfunction is the loss of the normal bidirectional functional relationship that exists between the heart and the lungs [1,2]. It can range in severity from subclinical (and only manifesting under stress) to fatal, with a timeline that can be acute, chronic, or acute on chronic. Cardiopulmonary dysfunction can be the consequence of single-organ (heart or lung) disease, systemic disease (eg, sepsis) [3,4], or iatrogenic (eg, medications or mechanical ventilation) [5,6].

The investigation of cardiopulmonary dysfunction requires an assessment of the complex heart-lung relationship. The 2 primary interfaces where these organs interact are the right heart-pulmonary circulation relationship (often referred to as “coupling”) and the alveolus-pulmonary capillary relationship [7]. Coupling denotes the relationship between the pulsatile pump of the right ventricle and its opposing arterial vascular load (ie, pulmonary arterial circulation) [8]. As one component of this relationship changes (eg, the increase in arterial stiffness seen in aging due to loss of vessel elasticity) [9], the other responds (eg, with ventricular remodeling) to maintain normal coupling, giving optimal cardiac mechanics and cardiopulmonary efficiency at rest [10].

The alveolus-pulmonary capillary relationship has pressure as its currency. In the healthy upright lung, the majority of ventilation and perfusion occurs at the lung base, where both the pulmonary arterial and pulmonary venous pressures exceed the alveolar gas pressure (West zone 3) [11]. This facilitates constant blood flow past the alveolus giving uninterrupted gas exchange—in normal physiological conditions the alveolar pressure does not affect blood flow through the pulmonary circulation [11,12]. Lung hyperinflation can cause compression of the alveolar vessels leading to raised pulmonary vascular pressures, an increase in pulmonary blood transit time, myocardial hypoperfusion, and biventricular systolic dysfunction [13-16]. If there is concurrent right heart dysfunction, there may also be reduced pulmonary vascular blood flow, further worsening this process [15]. Patients with airway disease have a predilection for progressive (dynamic) hyperinflation during exercise, a condition characterized by increasing end-expiratory lung volume. This increase in volume within the fixed thoracic cage leads to an increase in the intra-alveolar pressure, with higher degrees of dynamic hyperinflation related to a poorer cardiac output during the activity [17].

In the outpatient setting, the investigation of cardiopulmonary dysfunction often involves a series of single-organ assessment tools such as computed tomography imaging of the lung, complex lung function testing, and transthoracic echocardiography. These single-organ, static investigations provide little insight into cardiopulmonary performance when under physiological (eg, exercise) or pathological (eg, sepsis) stress [18,19]. Current commonly used functional testing modalities such as stress echocardiography can provide additional information and have a role in the investigation of specific conditions in patients with the appropriate pretest probability—for example, assessing for coronary artery disease in patients with anginal symptoms [5]. However, this test is not capable of assessing the intricacies of cardiopulmonary dysfunction. A cardiopulmonary exercise test (CPET) provides integrative information about cardiac, respiratory, and metabolic responses to exercise and is considered the gold standard in the integrative assessment of cardiorespiratory function [20]. Peak oxygen consumption (VO2peak) measured by CPET is the key measure of cardiopulmonary fitness [21], has a stronger relationship with all-cause mortality than many common chronic diseases [22], and can be improved with low-burden lifestyle interventions [23]. Despite this, CPET also has limitations, most notably that it provides no anatomical information about cardiac function under an increasing workload. As a result, conditions with significant prognostic implications including exercise-induced pulmonary hypertension [24] or exercise-induced diastolic function [25] may go unnoticed.

Recently the combination of cardiopulmonary exercise test-stress echocardiography (CPET-SE) has been used to explore cardiopulmonary dysfunction. Studies have explored the role of CPET-SE in exploring conditions such as dyspnea [26], heart failure [27,28], contractile reserve [29], and left atrial dysfunction [30]. While the application is broad, the indications for this test combination remain undefined, and there is no consensus regarding the optimal protocol for the exercise and imaging components. Similarly, any additional prognostic benefits over traditional testing are undefined.

Given the complexity involved in investigating cardiopulmonary dysfunction, the breadth of applications for CPET-SE, and the lack of standardization for the test, we propose a scoping review to assess the literature surrounding the use of CPET-SE in investigating cardiopulmonary dysfunction in the outpatient setting. This will include an exploration of its impact on shaping the diagnostic and management processes of cardiopulmonary dysfunction. This scoping review will provide shape to the literature regarding this combined testing modality and provide an evidence base for future research.

A preliminary search of MEDLINE, the Cochrane Database of Systematic Reviews, and Joanna Briggs Institute (JBI) Evidence Synthesis was conducted and no current or underway systematic reviews or scoping reviews on the topic were identified.

## Methods

### Overview

This scoping review will be conducted in accordance with the JBI methodology for scoping reviews [31], based on earlier work from Arksey and O’Malley [32]. It will follow the process of (1) identifying the research question; (2) identifying the relevant studies; (3) defining criteria for study inclusion; (4) data charting; (5) collation, summarizing, and reporting of results; and (6) peer feedback [29]. The PRISMA-ScR (Preferred Reporting Items for Systematic Reviews and Meta-Analyses Extension for Scoping Reviews) [33] checklist will be followed.

#### Identification of the Research Question

The primary question of this scoping review is “What is the role of simultaneous cardiopulmonary exercise testing and echocardiography in investigating adult outpatients with symptoms of cardiopulmonary dysfunction?” Associated subquestions for this review are “What are the primary pathologies in which simultaneous cardiopulmonary exercise testing and echocardiography has been used?” and “Is there any evidence that CPET-SE provides additional diagnostic benefit over traditional testing?”

#### Identification of the Relevant Studies

##### Overview

The Population, Concept, Context (PCC) framework was used to scaffold the main components of the research question.

##### Population, Concept, and Context

Population and concept have been combined in this scoping review as they are interlinked, that is, it is inclusive of all patients undergoing this particular combined investigatory modality.

All studies involving patients aged 18 years or older with either known cardiopulmonary disease or symptoms suggestive of cardiopulmonary dysfunction who underwent a simultaneous transthoracic echocardiogram and CPET will be included. There will be no limitations regarding specific disease states or symptom burden.

To best capture the potential breadth of the literature, all studies involving simultaneous echocardiography and cardiopulmonary exercise testing will be included. The minimum data set required for the CPET is continuous gas exchange data, continuous cardiac monitoring, and either a stationary bike or treadmill ergometer. This is in line with accepted clinical practice [34].

The outcomes of interest are the range of contexts in which the combined investigation technique has been applied and the overall assessment of its use in providing clinically relevant information.

##### Sources

This scoping review will consider the following study designs: randomized and nonrandomized controlled trials, observational studies (retrospective and prospective), and descriptive studies including case series and reports. Systematic reviews that meet the inclusion criteria will also be included.

A hand search of all studies included in the final review will be performed to identify additional literature that may not have been uncovered in the initial database search. Published abstracts and conference proceedings will be considered if they meet the inclusion criteria. Consideration will also be given to grey literature (including expert opinion pieces) if they contain sufficient detail to meet the inclusion criteria. Such data will be identified through discussion with an existing local and international network of experts in the field.

##### Study or Source of Evidence Selection

All identified citations will be collated via the online software Covidence [35]. EndNote (version 20.3; Clarivate Analytics) will be used as the reference management software. The title and abstract screening will be performed by 2 independent reviewers to determine relevance and suitability using the inclusion criteria.

Studies identified as relevant will undergo full-text assessment by 2 independent reviewers. Appropriate studies will undergo data extraction. Studies deemed inappropriate will have the rationale for such recorded and reported in the scoping review.

Disagreements at either title-abstract or full-text screening will be resolved through the use of a third independent reviewer. The results of the search and the study inclusion process will be reported in full in the final scoping review and presented in a PRISMA-ScR flow diagram [33].

#### Study Inclusion Criteria

The search strategy will be constructed with assistance from an experienced academic librarian. An initial limited search of MEDLINE and Scopus was performed to identify relevant keywords from titles and abstracts. These index terms were used to develop the complete search strategy for MEDLINE, Scopus, Embase, and the Cochrane Library (including reviews, protocols, and trials; see Multimedia Appendix 1). The search strategy, including all identified keywords and index terms, will be adapted for each included database and information source. The search strategy deliberately does not reference inpatient or outpatient to ensure that the search results have an appropriate breadth. Only studies published in English will be included. No date restrictions are in place, that is, the databases will be searched from inception to the search date.

#### Data Charting

Data extraction will be performed by 2 independent reviewers using a customized data extraction tool based on the JBI template [31] (see Multimedia Appendix 2 for the draft data extraction tool). This tool will be piloted using the first 10% of the included full-text studies. After this period the tool will be reevaluated and modified as appropriate to ensure collection of all relevant data. If necessary, this modified (final) data extraction tool will be used for all included full-text studies including the cohort used in the pilot.

Any disagreements with the data charting process will be resolved through discussion, or where necessary, by a third independent reviewer. Where there is missing data, the authors of the original manuscripts will be contacted for additional data or clarification. A critical appraisal of the articles included in the full-text analysis will not be performed.

#### Result in Collation, Summary, and Reporting

The PRISMA-ScR [33] checklist will be used to guide collation and reporting. The extracted data will be presented predominately in tabular format, in line with the data extraction tool and the review questions of this scoping review (Multimedia Appendix 3). It is expected that some data (specifically diagnostic categories and symptom burden) will also be presented diagrammatically to aid clarity. A narrative summary will accompany the tabulated results and will describe how the results relate to the objective and research questions of this scoping review.

#### Validation, Consultation, and Feedback

Local experts on echocardiography, cardiopulmonary disease, and exercise testing not involved in the authorship of this protocol will be consulted at specific points in this process, including during stages 3 and 5. An academic librarian will be used during stage 2 to ensure the search is thorough and relevant.

### Ethical Considerations

Ethics approval has not been sought for this review as data are being collected from published literature.

## Results

This scoping review is expected to yield results by late 2023 or early 2024. These results will be presented within the local hospital network by the authorship group to inform current clinical practice and provide an evidence base for future studies using this combined modality. The results of this scoping review will also be presented in article format and submitted for publication in a relevant peer-reviewed journal.

## Discussion

### Principal Findings

We seek to explore the application and outcomes of a combination of CPET-SE in clinical practice. To the best of our knowledge, this is the first exploratory review on this subject and will be a comprehensive description of the literature.

Advanced functional testing (including CPET) provides data that are more representative of an individual’s ability to tolerate a stressor (eg, surgery) or it can induce the symptom (eg, exercise intolerance) of a patient while simultaneously capturing a comprehensive data set for thorough investigation. Combining this with real-time imaging such as echocardiography adds another layer of information [26,27]. Individually there are international guidelines for these tests [20,36,37], but how to best combine them is not clear.

There are significant potential advantages with the CPET-SE; however, the literature appears heterogeneous across testing or acquisition protocols and inconsistent in reporting results making interpretation challenging for clinicians. This scoping review can serve as part of the groundwork required for future guidelines regarding simultaneous CPET-SE.

Strengths of this protocol include the use of a structured, multi-database literature search with few constraints; the use of an established scoping review framework; and the use of independent expert consultation throughout the process.

### Limitations

There are 2 primary limitations to this scoping review. The first is the risk of missing relevant studies in the search steps. Multiple databases relevant to medical literature are being included, and the search terms include both whole words and conventional acronyms for CPET, there is a risk that there are additional acronyms or abbreviations used internationally that are unknown to the authors of the scoping review and therefore the literature may be missed. Limiting search results to articles published in English is the second limitation.

### Conclusions

The role of exercise testing in clinical practice is expanding, and with this expansion comes novel testing regimens such as CPET-SE. By providing a summary of the application of combined CPET and echocardiography in current practice we will have the base from which a unified, comprehensive, and standardized testing approach can be proposed including indications, testing procedures, and reporting protocols. This will benefit clinicians (by providing an evidence base on which they can structure their testing), researchers (by giving a degree of data homogeneity and allowing more rigorous statistical analysis), and ultimately patients.

## Appendix

Multimedia Appendix 1 Search strategy.

Multimedia Appendix 2 Data extraction instrument.

Multimedia Appendix 3 Data presentation instrument.

## Abbreviations

CPET: cardiopulmonary exercise test
CPET-SE: cardiopulmonary exercise test-stress echocardiography
JBI: Joanna Briggs Institute
PCC: Population, Concept, Context
PRISMA-ScR: Preferred Reporting Items for Systematic Reviews and Meta-Analyses Extension for Scoping Reviews

## Data Availability

Data sharing is not applicable to this study as no data sets were generated or analyzed during this study.
