# Supplementary material for: Simultaneous Cardiopulmonary Exercise Testing and Echocardiography for Investigation of Cardiopulmonary Dysfunction in Outpatients: Protocol for a Scoping Review
Source: JMIR Res Protoc. 2024 Feb 12;13:e52076. doi: 10.2196/52076 (PMC10897791; doi:10.2196/52076)
Supplement: Multimedia Appendix 1 [file resprot_v13i1e52076_app1.docx]

The search terms for Medline, Scopus and Embase are:

( TITLE-ABS-KEY ( "cardiopulmonary exercise test*" OR cpet OR cpex OR cpx ) AND TITLE-ABS-KEY ( "stress echo*" OR "exercise stress echo*" OR se OR ese ) )

Limits: English language only.

No date limit will be set.

The search terms for the Cochrane Library (including reviews, protocols and trials) are:

#1. “cardiopulmonary exercise test*” OR cpet OR cpex OR cpx

#2. “stress echo*” OR “exercise stress echo*” OR se OR ese

#3. #1 and #2
