# Supplementary material for: Simultaneous Cardiopulmonary Exercise Testing and Echocardiography for Investigation of Cardiopulmonary Dysfunction in Outpatients: Protocol for a Scoping Review
Source: JMIR Res Protoc. 2024 Feb 12;13:e52076. doi: 10.2196/52076 (PMC10897791; doi:10.2196/52076)
Supplement: Multimedia Appendix 2 [file resprot_v13i1e52076_app2.docx]

| Evidence source, details and characteristics | |
| --- | --- |
| Citation details (e.g. author/s, date, title, journal, volume, issue, pages) |  |
| Country |  |
| Type of study/literature (e.g. randomised controlled trial, observational trial, retrospective/prospective, etc) |  |
| Results extracted from source of evidence (in relation to the concept of the scoping review) | |
| Patient characteristics   - Number of patients - Mean age - Gender - Cardiopulmonary diagnosis |  |
| Implications on patient care (diagnostics or management) |  |
| Was the combined modality deemed diagnostically useful by the authors |  |
| Recommendations for utilisation/further investigation by the authors |  |
