# Supplementary material for: Simultaneous Cardiopulmonary Exercise Testing and Echocardiography for Investigation of Cardiopulmonary Dysfunction in Outpatients: Protocol for a Scoping Review
Source: JMIR Res Protoc. 2024 Feb 12;13:e52076. doi: 10.2196/52076 (PMC10897791; doi:10.2196/52076)
Supplement: Multimedia Appendix 3 [file resprot_v13i1e52076_app3.docx]

| **Study details** | | | | **Study characteristics** | | | | **Outcomes** | | |
| --- | --- | --- | --- | --- | --- | --- | --- | --- | --- | --- |
| Author | Year | Country | Study design | Patient number | Mean age | Gender | diagnosis | Effect on patient care | Was the combination deemed useful | Recommendations for further investigation |
